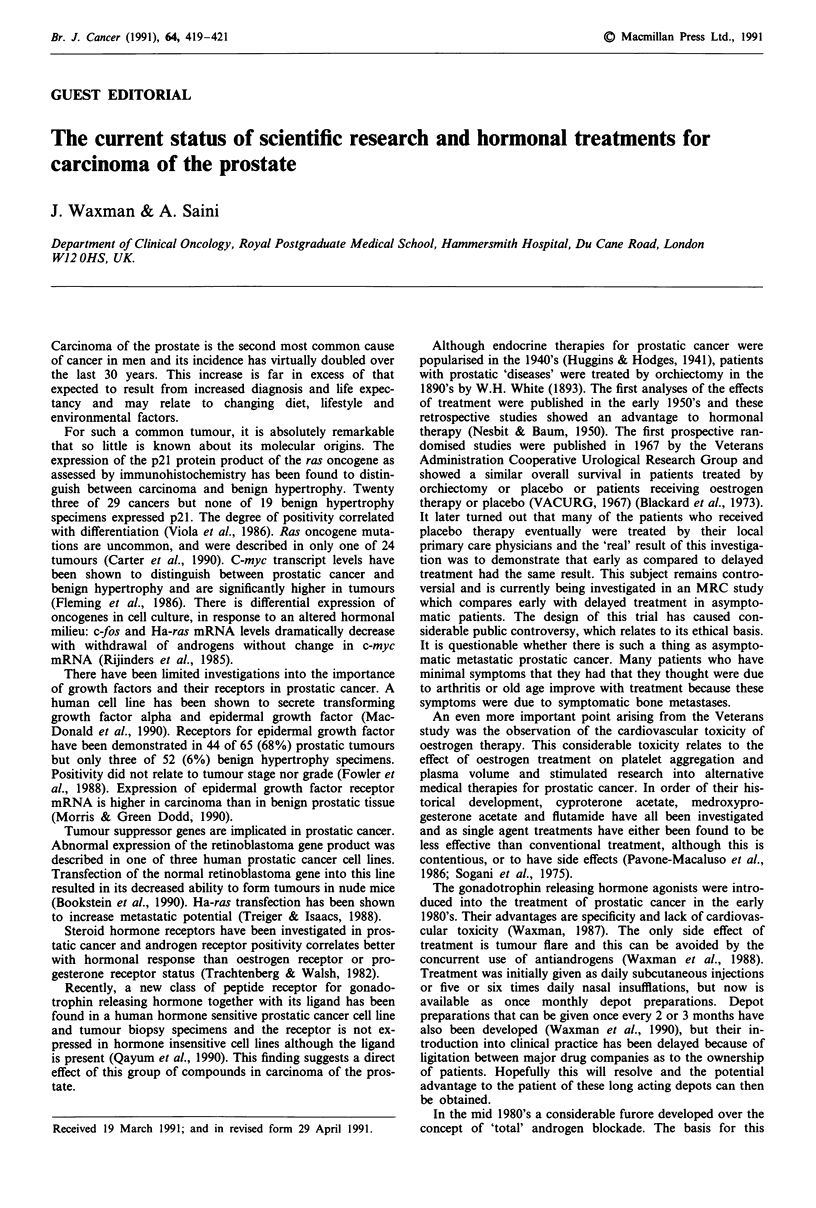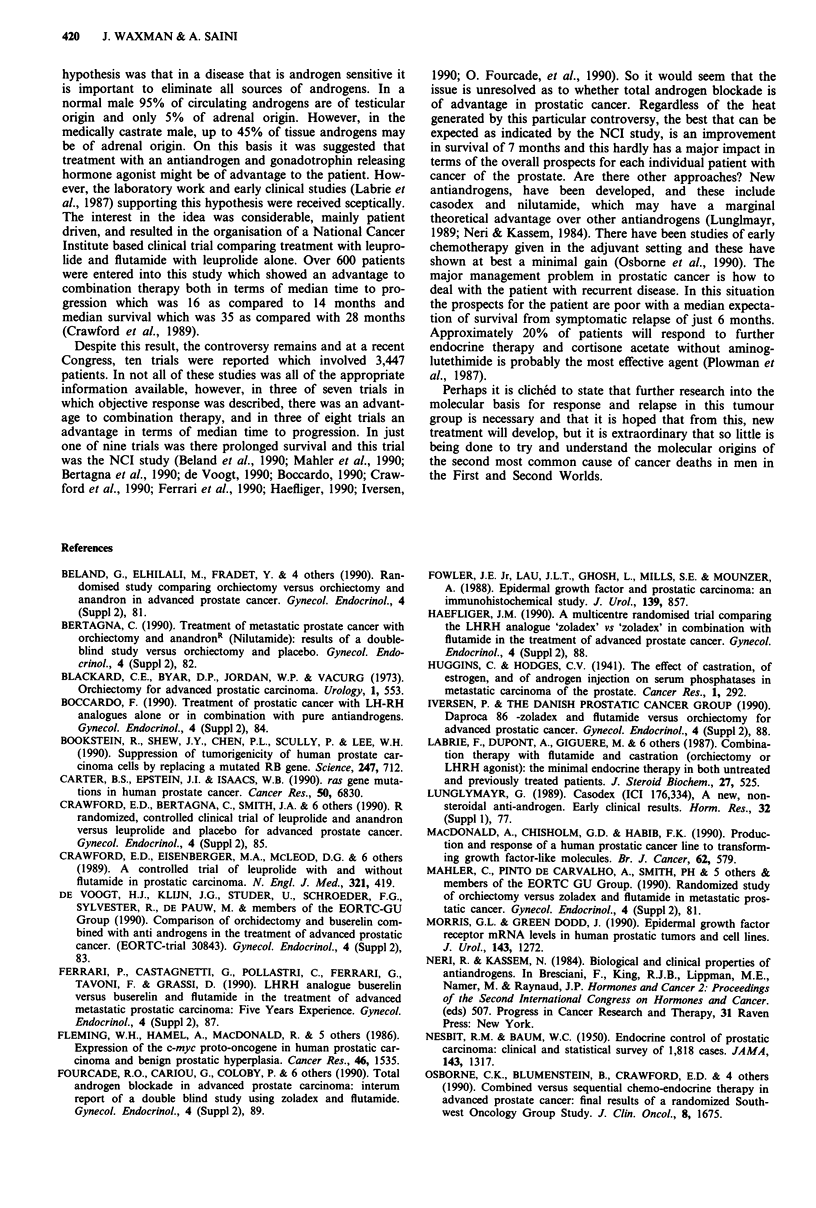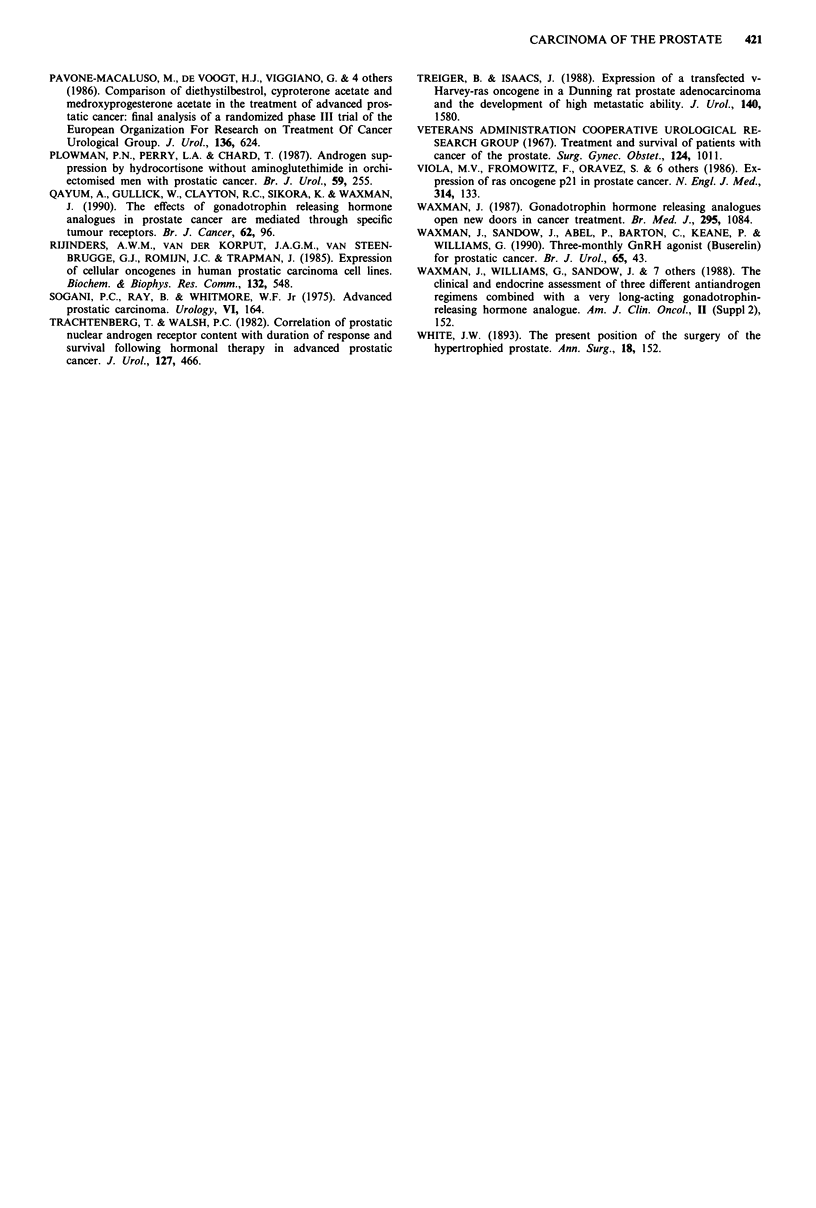# The current status of scientific research and hormonal treatments for carcinoma of the prostate.

**DOI:** 10.1038/bjc.1991.324

**Published:** 1991-09

**Authors:** J. Waxman, A. Saini


*Br. J. Cancer* (1991), **64**, 419–421 © Macmillan Press Ltd., 1991

GUEST EDITORIAL

### The current status of scientific research and hormonal treatments for carcinoma of the prostate

J. Waxman & A. Saini

*Department of Clinical Oncology, Royal Postgraduate Medical School, Hammersmith Hospital, Du Cane Road, London W12 0HS, UK.*

Carcinoma of the prostate is the second most common cause of cancer in men and its incidence has virtually doubled over the last 30 years. This increase is far in excess of that expected to result from increased diagnosis and life expectancy and may relate to changing diet, lifestyle and environmental factors.

For such a common tumour, it is absolutely remarkable that so little is known about its molecular origins. The expression of the p21 protein product of the *ras* oncogene as assessed by immunohistochemistry has been found to distinguish between carcinoma and benign hypertrophy. Twenty three of 29 cancers but none of 19 benign hypertrophy specimens expressed p21. The degree of positivity correlated with differentiation (Viola *et al.*, 1986). *Ras* oncogene mutations are uncommon, and were described in only one of 24 tumours (Carter *et al.*, 1990). C-*myc* transcript levels have been shown to distinguish between prostatic cancer and benign hypertrophy and are significantly higher in tumours (Fleming *et al.*, 1986). There is differential expression of oncogenes in cell culture, in response to an altered hormonal milieu: c-*fos* and Ha-*ras* mRNA levels dramatically decrease with withdrawal of androgens without change in c-*myc* mRNA (Rijinders *et al.*, 1985).

There have been limited investigations into the importance of growth factors and their receptors in prostatic cancer. A human cell line has been shown to secrete transforming growth factor alpha and epidermal growth factor (MacDonald *et al.*, 1990). Receptors for epidermal growth factor have been demonstrated in 44 of 65 (68%) prostatic tumours but only three of 52 (6%) benign hypertrophy specimens. Positivity did not relate to tumour stage nor grade (Fowler *et al.*, 1988). Expression of epidermal growth factor receptor mRNA is higher in carcinoma than in benign prostatic tissue (Morris & Green Dodd, 1990).

Tumour suppressor genes are implicated in prostatic cancer. Abnormal expression of the retinoblastoma gene product was described in one of three human prostatic cancer cell lines. Transfection of the normal retinoblastoma gene into this line resulted in its decreased ability to form tumours in nude mice (Bookstein *et al.*, 1990). Ha-*ras* transfection has been shown to increase metastatic potential (Treiger & Isaacs, 1988).

Steroid hormone receptors have been investigated in prostatic cancer and androgen receptor positivity correlates better with hormonal response than oestrogen receptor or progesterone receptor status (Trachtenberg & Walsh, 1982).

Recently, a new class of peptide receptor for gonadotrophin releasing hormone together with its ligand has been found in a human hormone sensitive prostatic cancer cell line and tumour biopsy specimens and the receptor is not expressed in hormone insensitive cell lines although the ligand is present (Qayum *et al.*, 1990). This finding suggests a direct effect of this group of compounds in carcinoma of the prostate.

Received 19 March 1991; and in revised form 29 April 1991.

Although endocrine therapies for prostatic cancer were popularised in the 1940's (Huggins & Hodges, 1941), patients with prostatic 'diseases' were treated by orchiectomy in the 1890's by W.H. White (1893). The first analyses of the effects of treatment were published in the early 1950's and these retrospective studies showed an advantage to hormonal therapy (Nesbit & Baum, 1950). The first prospective randomised studies were published in 1967 by the Veterans Administration Cooperative Urological Research Group and showed a similar overall survival in patients treated by orchiectomy or placebo or patients receiving oestrogen therapy or placebo (VACURG, 1967) (Blackard *et al.*, 1973). It later turned out that many of the patients who received placebo therapy eventually were treated by their local primary care physicians and the 'real' result of this investigation was to demonstrate that early as compared to delayed treatment had the same result. This subject remains controversial and is currently being investigated in an MRC study which compares early with delayed treatment in asymptomatic patients. The design of this trial has caused considerable public controversy, which relates to its ethical basis. It is questionable whether there is such a thing as asymptomatic metastatic prostatic cancer. Many patients who have minimal symptoms that they had that they thought were due to arthritis or old age improve with treatment because these symptoms were due to symptomatic bone metastases.

An even more important point arising from the Veterans study was the observation of the cardiovascular toxicity of oestrogen therapy. This considerable toxicity relates to the effect of oestrogen treatment on platelet aggregation and plasma volume and stimulated research into alternative medical therapies for prostatic cancer. In order of their historical development, cyproterone acetate, medroxyprogesterone acetate and flutamide have all been investigated and as single agent treatments have either been found to be less effective than conventional treatment, although this is contentious, or to have side effects (Pavone-Macaluso *et al.*, 1986; Sogani *et al.*, 1975).

The gonadotrophin releasing hormone agonists were introduced into the treatment of prostatic cancer in the early 1980's. Their advantages are specificity and lack of cardiovascular toxicity (Waxman, 1987). The only side effect of treatment is tumour flare and this can be avoided by the concurrent use of antiandrogens (Waxman *et al.*, 1988). Treatment was initially given as daily subcutaneous injections or five or six times daily nasal insufflations, but now is available as once monthly depot preparations. Depot preparations that can be given once every 2 or 3 months have also been developed (Waxman *et al.*, 1990), but their introduction into clinical practice has been delayed because of ligitation between major drug companies as to the ownership of patients. Hopefully this will resolve and the potential advantage to the patient of these long acting depots can then be obtained.

In the mid 1980's a considerable furore developed over the concept of 'total' androgen blockade. The basis for this hypothesis was that in a disease that is androgen sensitive it is important to eliminate all sources of androgens. In a normal male 95% of circulating androgens are of testicular origin and only 5% of adrenal origin. However, in the medically castrate male, up to 45% of tissue androgens may be of adrenal origin. On this basis it was suggested that treatment with an antiandrogen and gonadotrophin releasing hormone agonist might be of advantage to the patient. However, the laboratory work and early clinical studies (Labrie *et al.*, 1987) supporting this hypothesis were received sceptically. The interest in the idea was considerable, mainly patient driven, and resulted in the organisation of a National Cancer Institute based clinical trial comparing treatment with leuprolide and flutamide with leuprolide alone. Over 600 patients were entered into this study which showed an advantage to combination therapy both in terms of median time to progression which was 16 as compared to 14 months and median survival which was 35 as compared with 28 months (Crawford *et al.*, 1989).

Despite this result, the controversy remains and at a recent Congress, ten trials were reported which involved 3,447 patients. In not all of these studies was all of the appropriate information available, however, in three of seven trials in which objective response was described, there was an advantage to combination therapy, and in three of eight trials an advantage in terms of median time to progression. In just one of nine trials was there prolonged survival and this trial was the NCI study (Beland *et al.*, 1990; Mahler *et al.*, 1990; Bertagna *et al.*, 1990; de Voogt, 1990; Boccardo, 1990; Crawford *et al.*, 1990; Ferrari *et al.*, 1990; Haefliger, 1990; Iversen, 1990; O. Fourcade, *et al.*, 1990). So it would seem that the issue is unresolved as to whether total androgen blockade is of advantage in prostatic cancer. Regardless of the heat generated by this particular controversy, the best that can be expected as indicated by the NCI study, is an improvement in survival of 7 months and this hardly has a major impact in terms of the overall prospects for each individual patient with cancer of the prostate. Are there other approaches? New antiandrogens, have been developed, and these include casodex and nilutamide, which may have a marginal theoretical advantage over other antiandrogens (Lunglmayr, 1989; Neri & Kassem, 1984). There have been studies of early chemotherapy given in the adjuvant setting and these have shown at best a minimal gain (Osborne *et al.*, 1990). The major management problem in prostatic cancer is how to deal with the patient with recurrent disease. In this situation the prospects for the patient are poor with a median expectation of survival from symptomatic relapse of just 6 months. Approximately 20% of patients will respond to further endocrine therapy and cortisone acetate without aminoglutethimide is probably the most effective agent (Plowman *et al.*, 1987).

Perhaps it is clichéd to state that further research into the molecular basis for response and relapse in this tumour group is necessary and that it is hoped that from this, new treatment will develop, but it is extraordinary that so little is being done to try and understand the molecular origins of the second most common cause of cancer deaths in men in the First and Second Worlds.

### References

BELAND, G., ELHILALI, M., FRADET, Y. & 4 others (1990). Randomised study comparing orchiectomy versus orchiectomy and anandron in advanced prostate cancer. *Gynecol. Endocrinol.*, **4** (Suppl 2), 81.

BERTAGNA, C. (1990). Treatment of metastatic prostate cancer with orchiectomy and anandron[R] (Nilutamide): results of a double-blind study versus orchiectomy and placebo. *Gynecol. Endocrinol.*, **4** (Suppl 2), 82.

BLACKARD, C.E., BYAR, D.P., JORDAN, W.P. & VACURG (1973). Orchiectomy for advanced prostatic carcinoma. *Urology*, **1**, 553.

BOCCARDO, F. (1990). Treatment of prostatic cancer with LH-RH analogues alone or in combination with pure antiandrogens. *Gynecol. Endocrinol.*, **4** (Suppl 2), 84.

BOOKSTEIN, R., SHEW, J.Y., CHEN, P.L., SCULLY, P. & LEE, W.H. (1990). Suppression of tumorigenicity of human prostate carcinoma cells by replacing a mutated RB gene. *Science*, **247**, 712.

CARTER, B.S., EPSTEIN, J.I. & ISAACS, W.B. (1990). *ras* gene mutations in human prostate cancer. *Cancer Res.*, **50**, 6830.

CRAWFORD, E.D., BERTAGNA, C., SMITH, J.A. & 6 others (1990). R randomized, controlled clinical trial of leuprolide and anandron versus leuprolide and placebo for advanced prostate cancer. *Gynecol. Endocrinol.*, **4** (Suppl 2), 85.

CRAWFORD, E.D., EISENBERGER, M.A., MCLEOD, D.G. & 6 others (1989). A controlled trial of leuprolide with and without flutamide in prostatic carcinoma. *N. Engl. J. Med.*, **321**, 419.

DE VOOGT, H.J., KLIJN, J.G., STUDER, U., SCHROEDER, F.G., SYLVESTER, R., DE PAUW, M. & members of the EORTC-GU Group (1990). Comparison of orchidectomy and buserelin combined with anti androgens in the treatment of advanced prostatic cancer. (EORTC-trial 30843). *Gynecol. Endocrinol.*, **4** (Suppl 2), 83.

FERRARI, P., CASTAGNETTI, G., POLLASTRI, C., FERRARI, G., TAVONI, F. & GRASSI, D. (1990). LHRH analogue buserelin versus buserelin and flutamide in the treatment of advanced metastatic prostatic carcinoma: Five Years Experience. *Gynecol. Endocrinol.*, **4** (Suppl 2), 87.

FLEMING, W.H., HAMEL, A., MACDONALD, R. & 5 others (1986). Expression of the c-*myc* proto-oncogene in human prostatic carcinoma and benign prostatic hyperplasia. *Cancer Res.*, **46**, 1535.

FOURCADE, R.O., CARIOU, G., COLOBY, P. & 6 others (1990). Total androgen blockade in advanced prostate carcinoma: interum report of a double blind study using zoladex and flutamide. *Gynecol. Endocrinol.*, **4** (Suppl 2), 89.

FOWLER, J.E. Jr, LAU, J.L.T., GHOSH, L., MILLS, S.E. & MOUNZER, A. (1988). Epidermal growth factor and prostatic carcinoma: an immunohistochemical study. *J. Urol.*, **139**, 857.

HAEFLIGER, J.M. (1990). A multicentre randomised trial comparing the LHRH analogue 'zoladex' *vs* 'zoladex' in combination with flutamide in the treatment of advanced prostate cancer. *Gynecol. Endocrinol.*, **4** (Suppl 2), 88.

HUGGINS, C. & HODGES, C.V. (1941). The effect of castration, of estrogen, and of androgen injection on serum phosphatases in metastatic carcinoma of the prostate. *Cancer Res.*, **1**, 292.

IVERSEN, P. & THE DANISH PROSTATIC CANCER GROUP (1990). Daproca 86 -zoladex and flutamide versus orchiectomy for advanced prostatic cancer. *Gynecol. Endocrinol.*, **4** (Suppl 2), 88.

LABRIE, F., DUPONT, A., GIGUERE, M. & 6 others (1987). Combination therapy with flutamide and castration (orchiectomy or LHRH agonist): the minimal endocrine therapy in both untreated and previously treated patients. *J. Steroid Biochem.*, **27**, 525.

LUNGLYMAYR, G. (1989). Casodex (ICI 176,334), A new, non-steroidal anti-androgen. Early clinical results. *Horm. Res.*, **32** (Suppl 1), 77.

MACDONALD, A., CHISHOLM, G.D. & HABIB, F.K. (1990). Production and response of a human prostatic cancer line to transforming growth factor-like molecules. *Br. J. Cancer*, **62**, 579.

MAHLER, C., PINTO DE CARVALHO, A., SMITH, PH & 5 others & members of the EORTC GU Group. (1990). Randomized study of orchiectomy versus zoladex and flutamide in metastatic prostatic cancer. *Gynecol. Endocrinol.*, **4** (Suppl 2), 81.

MORRIS, G.L. & GREEN DODD, J. (1990). Epidermal growth factor receptor mRNA levels in human prostatic tumors and cell lines. *J. Urol.*, **143**, 1272.

NERI, R. & KASSEM, N. (1984). Biological and clinical properties of antiandrogens. In Bresciani, F., King, R.J.B., Lippman, M.E., Namer, M. & Raynaud, J.P. *Hormones and Cancer 2: Proceedings of the Second International Congress on Hormones and Cancer.* (eds) 507. Progress in Cancer Research and Therapy, **31** Raven Press: New York.

NESBIT, R.M. & BAUM, W.C. (1950). Endocrine control of prostatic carcinoma: clinical and statistical survey of 1,818 cases. *JAMA*, **143**, 1317.

OSBORNE, C.K., BLUMENSTEIN, B., CRAWFORD, E.D. & 4 others (1990). Combined versus sequential chemo-endocrine therapy in advanced prostate cancer: final results of a randomized South-west Oncology Group Study. *J. Clin. Oncol.*, **8**, 1675.

PAVONE-MACALUSO, M., DE VOOGT, H.J., VIGGIANO, G. & 4 others (1986). Comparison of diethystilbestrol, cyproterone acetate and medroxyprogesterone acetate in the treatment of advanced prostatic cancer: final analysis of a randomized phase III trial of the European Organization For Research on Treatment Of Cancer Urological Group. *J. Urol.*, **136**, 624.

PLOWMAN, P.N., PERRY, L.A. & CHARD, T. (1987). Androgen suppression by hydrocortisone without aminoglutethimide in orchiectomised men with prostatic cancer. *Br. J. Urol.*, **59**, 255.

QAYUM, A., GULLICK, W., CLAYTON, R.C., SIKORA, K. & WAXMAN, J. (1990). The effects of gonadotrophin releasing hormone analogues in prostate cancer are mediated through specific tumour receptors. *Br. J. Cancer*, **62**, 96.

RIJINDERS, A.W.M., VAN DER KORPUT, J.A.G.M., VAN STEENBRUGGE, G.J., ROMIJN, J.C. & TRAPMAN, J. (1985). Expression of cellular oncogenes in human prostatic carcinoma cell lines. *Biochem. & Biophys. Res. Comm.*, **132**, 548.

SOGANI, P.C., RAY, B. & WHITMORE, W.F. Jr (1975). Advanced prostatic carcinoma. *Urology*, **VI**, 164.

TRACHTENBERG, T. & WALSH, P.C. (1982). Correlation of prostatic nuclear androgen receptor content with duration of response and survival following hormonal therapy in advanced prostatic cancer. *J. Urol.*, **127**, 466.

TREIGER, B. & ISAACS, J. (1988). Expression of a transfected v-Harvey-ras oncogene in a Dunning rat prostate adenocarcinoma and the development of high metastatic ability. *J. Urol.*, **140**, 1580.

VETERANS ADMINISTRATION COOPERATIVE UROLOGICAL RESEARCH GROUP (1967). Treatment and survival of patients with cancer of the prostate. *Surg. Gynec. Obstet.*, **124**, 1011.

VIOLA, M.V., FROMOWITZ, F., ORAVEZ, S. & 6 others (1986). Expression of ras oncogene p21 in prostate cancer. *N. Engl. J. Med.*, **314**, 133.

WAXMAN, J. (1987). Gonadotrophin hormone releasing analogues open new doors in cancer treatment. *Br. Med. J.*, **295**, 1084.

WAXMAN, J., SANDOW, J., ABEL, P., BARTON, C., KEANE, P. & WILLIAMS, G. (1990). Three-monthly GnRH agonist (Buserelin) for prostatic cancer. *Br. J. Urol.*, **65**, 43.

WAXMAN, J., WILLIAMS, G., SANDOW, J. & 7 others (1988). The clinical and endocrine assessment of three different antiandrogen regimens combined with a very long-acting gonadotrophin-releasing hormone analogue. *Am. J. Clin. Oncol.*, **II** (Suppl 2), 152.

WHITE, J.W. (1893). The present position of the surgery of the hypertrophied prostate. *Ann. Surg.*, **18**, 152.